# Neurological complications following mRNA COVID-19 vaccination: Oral and lower limb paresthesia. A case discussion and literature review

**DOI:** 10.4317/jced.60891

**Published:** 2023-10-01

**Authors:** Carmen López-Carriches, Mª Isabel Leco-Berrocal, Ricardo Bahram-Taheri, Carlos Cardona-Moreno, Jorge Cortés-Bretón-Brinkmann, Luis-Alberto Moreno-López

**Affiliations:** 1 Associate Professor, Department of Dental Clinic Specialties. School of Dentistry, Universidad Complutense de Madrid, Spain; 2 Assistant Professor, Department of Dental Clinical Specialties, School of Dentistry, Universidad Complutense de Madrid, Spain; 3 Doctor of Dental Surgery. DDS. Collaborator. School of Dentistry, Universidad Complutense de Madrid. Spain

## Abstract

Vaccines used in the coronavirus pandemic have reported some minor side effects such as pain at the injection site, headache, myalgia and fever. Also major neurological side effects have been experienced by some patients. We present the clinical case of a healthy woman who two weeks after being vaccinated with the third dose of Moderna COVID-19 Vaccine, began to feel numbness in mouth, both feet, legs, interscapular space, and hands. She was diagnosed with distal sensory polyneuropathy caused by the vaccine. Progressive improvement was seen. The patient did not require corticosteroid medication. We reviewed the literature to assess the frequency of this type of complication.

** Key words:**COVID-19, SARS-CoV-2, vaccine, vaccination, peripheral axonal neuropathy, transverse myelitis, oral manifestations.

## Introduction

The number of patients diagnosed with COVID-19 is still increasing with over 10% ending in death (1). Many of these have been related to increased age. Therefore massive vaccination of this population has had a vital importance to prevent this outcome.

The main mechanism of action of the FDA approved COVID-19 vaccines is messenger RNA (mRNA) vaccines (Comirnaty from Pfizer/BioNTech and Spikevax from Moderna). The mRNA encoding protein S is encapsulated in lipid nanoparticles. The nucleic acid vaccines introduce mRNA or DNA coding for SARS-CoV-2 spike protein into the cells, to induce cells to produce antibodies. These vaccines have reported an efficacy of up to 95% and are currently used in several countries (2).

Also, viral vector vaccines are used. These vaccines use a chemically weakened virus (e.g. adenovirus) to insert the code for SARS-CoV-2 antigens into the cells. Jcovden manufactured by Johnson and Johnson is produced with human adenovirus that carries protein S. Also, Vaxzevria, manufactured by Astrazeneca, includes non-replicative chimpanzee adenovirus which carries protein S (3).

Vaccines against COVID-19 have caused minor side effects such as pain and swelling at the injection site, headache, fever, or muscle aches. More important complications are also being reported, including neurological complications such as Bellʻs Palsy condition, Guillain-Barre syndrome, transverse myelitis, cerebral venous sinus thrombosis, acute disseminated encephalomyelitis, acute demyelinating polyneuropathy and reactivation of herpes zoster in many people (4,5).

## Case Report

Patient, 65-year-old woman, with no previous health problems, was vaccinated against COVID-19 with a third dose of Spikevax (Moderna). Two weeks after receiving the vaccine the patient arrived at a private dental clinic in Madrid with chief complaint of numbness in her mouth and both feet. Dental paresthesia enables patients to masticate with normality. This numbness progressively extended to the interscapular space and later to the hands. Muscle pain in the lower limbs and interscapular pain also were present.

Patient was referred to the neurologist. The neurological examination revealed vibratory hypoesthesia in the lower limbs, bilateral T2-T5 hypoesthesia and global hyperreflexia. The final diagnosis was distal and asymmetric sensory axonal polyneuropathy predominantly in the right lower limb, of moderate degree.

Electromyography (EMG) and electroneurography (ENG) test of the upper and lower limbs were performed. Electrical stimulation with percutaneous bipolar electrode was performed on the right median nerve from the elbow. Also, it was performed on the distal segment of the left median nerve, including its path through the carpal tunnel, on the distal segment of both Ulnar nerves, on both CPE nerves from the head of the fibula, on the posterior Tibial nerve right from the popliteal fossa, and both superficial peroneal and sural nerves.

Also, an ortho/antidromic sensory conduction study determined that there was a slight decrease in sensory conduction velocity in both sural nerves, with decreased amplitude of the evoked potentials on the right. The study showed normal values of sensory conduction of the right superficial peroneal nerve, with decreased amplitude of its evoked potentials.

There was a slight increase in the distal motor latency of the right median nerve, with normal motor conduction values in the rest of the explored path and preserved amplitude of its evoked potentials. Additionally, a decrease in the sensory conduction velocity of the distal segment of the right median nerve in its course through the wrist, with preserved amplitude of its evoked potentials was registered. Normal values of motor and sensory conduction were registered in the rest of the explored nerves, with conserved amplitude of their evoked potentials.

The neurophysiological study revealed an exclusively sensory polyneuropathy of the axonal type (demyelinating component), with a distal and asymmetric distribution, predominantly in the right lower limb.

In the upper limbs, data compatible with neuropathy due to entrapment of the distal segment of the right median nerve in its course through the wrist were recorded. Therefore, despite the symptoms, there was no polyneuropathy (PNP) in the upper limbs.

A full blood count revealed an increase in transaminases due to the destruction of myelin. The panel of antibodies against the SARS-CoV-2 virus, analyzed with multiplex immunoassay, gave the following results, in BAU units, which are an international standard developed by the WHO:

Anti-RBD IgG: greater than 4160 BAU/ml, the reference value being greater than or equal to 13 BAU/ml.

IgG Anti Spike 1: 7040 BAU7ml, being positive with greater than or equal to 22 BAU/ml.

IgG Anti Spike 2 results are 131 AU/ml, more than 10 times the reference value, which is greater than or equal to 10 AU/ml.

IgG Anti Nucleocapside level was less than 2.4 BAU / ml, which indicates that the patient has not had contact with the virus, since she does not appear after the vaccine. To be positive, the reference value must be greater than or equal to 24 BAU/ml.

**Figure 1:**
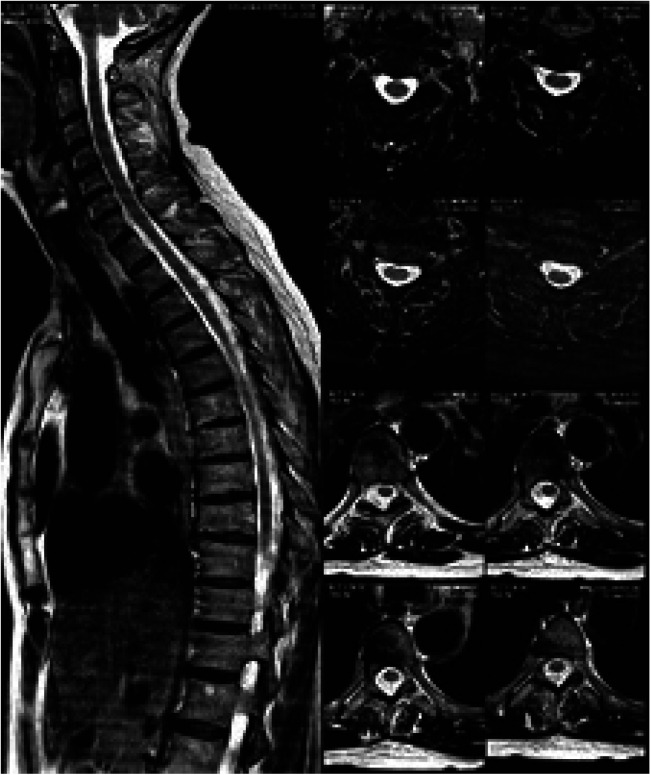
Cervical and dorsal magnetic resonance.

Cervical and dorsal magnetic resonance imaging was performed, (Fig. 1).

A normal craniocervical junction was observed. Also, there were no morphology alterations in the cervical or dorsal medullar cord that could explain neurological alterations.

Regarding the evolution of the patient, the first symptom that disappeared was the paresthesia of the teeth that lasted ten days. A month after, the muscular pain disappeared and the paresthesia in the legs got milder. Two months later, another ENG was sent. Progressive improvement was seen. The patient did not require corticosteroid medication.

## Discussion

The complications of our patient began with buccal paresthesia in all the teeth. Only one article has been identified which refers to oral hypoesthesia after vaccination (6).

Complications from vaccination against COVID-19 in the oral area are not very frequent and mostly affect the mucous membranes, such as inflammation of the lip or tongue. This could be related to anaphylaxis. (7) Also, mucositis has been related, producing moderate pain (8,9).

These complications are more frequent in females and older women. Additionally, mRNA-based COVID-19 vaccines produce more oral adverse effects (6). Ulcerative mucosal lesions, erythema, fissured tongue, and mucosal pain have been found in elderly patients (10,11).

There are several cases described of the appearance of oral lichen planus after vaccination or worsening of existing lichen lesions (12-15). A case of Oral Pemphigus(16) has been related to vaccination. Exacerbation of other oral mucosal autoimmune lesions have also been described (17)

In the analyzed literature, cases of complications have been found in the peripheral nervous system, especially Guillen Barre Syndrome, subacute axonal sensorimotor polyneuropathy, brachial plexopathy, chronic inflammatory demyelinating polyneuropathy (CIDP) (18,21).

## Conclusions

After the coronavirus vaccine, neurological complications can occur, both in the central and peripheral nervous systems, but these vaccines are essential in a pandemic like the one that has emerged, so the benefit outweighs the risk.
